# Preface

**DOI:** 10.1155/DTE.7.vii

**Published:** 2000

**Authors:** Yasumi Uchida

**Affiliations:** Clinical Physiology and Cardiovascular Center Toho University Hospital at Sakura Japan


*Diagnostic and Therapeutic Endoscopy*, Vol. 7, pp. vii–viii
Reprints available directly from the publisher
Photocopying permitted by license only

© 2000 OPA (Overseas Publishers Association) N.V.
Published by license under
the Harwood Academic Publishers imprint,
part of The Gordon and Breach Publishing Group.
Printed in Malaysia.

### Preface

In 1925, Dr. Kussmal in Germany devised a metallic tube attached to a lens inside for observation inside the stomach. As far as I know, this was the first endoluminal scope (endoscope) clinically applied.

On August 31, 1945, a big storm attacked the Kanto area in Japan. Trains stopped for several hours due to the storm and Dr. T. Uji, a surgeon of Tokyo University, and Mr. M. Sugiura of Olympus Co. happened to meet in the train. The concept of a new endoscope was discussed, which was the start of the development of the endoscope in Japan.

In December 1949, a flexible gastroscope 12mm in diameter with film and illumination at the distal tip was developed by the collaboration of Mr. M. Fukaomi and Mr. M. Maruyama. Anesthetized dogs were successfully examined with this endoscope.

Dr. T. Sakamoto used this scope for the first time clinically as a gastroscope. Thus, a flexible gastrofiberscope was developed and this has been the basic structure for bronchoscopes, cystoscopes, etc.

The endoscopy of the peripheral artery was first performed by Dr. S.M. Greenstone, however, the first percutaneous transluminal coronary angioscopy was performed many years after. It was due to the difficulty in the displacement of blood in the artery and the need for a more flexible and thinner endoscope.

The author and our colleagues of Olympus started to develop a new fiberscope for coronary use in 1976. Thrombosis and thrombolysis in the removed human coronary artery and the changes induced by balloon angioplasty were successfully observed by this endoscope in 1984.

Also, J.R. Spears observed coronary obstia by the use of a bronchoscope (1983) and coronary artery endoscopy was performed mainly intraoperatively by F. Litback (1985), F. Homebach (1986), T.A. Sanborn (1986), C.T. Sherman (1985), T. Vent (1987), etc.

Using fiberscopes specially designed for coronary arteries, the author observed percutaneously from proximal to distal coronary segments in patients with ischemic heart disease (1984, 1987). Observation of plaque disruption and thrombi in acute coronary syndromes were performed by V.H. Holer (1988), K. Mizuno (1991), P. den Heijer (1994), P.J. de Feyter (1995), J.A. Silva (1995), T. Thieme (1996), S. Waxman (1997) and others. Evaluation of coronary interventions by POBA was performed by Y. Uchida (1984), P. den Heijer (1994) and A. Itoh (1995), coronary interventions by laser by Y. Uchida (1987), F. Nakamura (1992) and A. Itoh (1993), and evaluation of coronary interventions by DCA, stent and cutting balloon were followed by many investigators.

Application of percutaneous coronary angioscopy was extended to the Kawasaki disease by T. Ishikawa, my colleague (1991) and for observation of CABG by the author (1994). Meanwhile, angioscopes of the monorail type without a balloon was devised by the author (1989) and those with a balloon by Baxter Co. and a 3-channel angioscopy balloon catheter by Y. Uchida (1994). Fiberscopes for coronary use are now reduced in outerdiameter down to 0.3mm. Thus, percutaneous angioscopy is now routinely performed in several institutions for examinations of underlying mechanisms of acute coronary syndromes, for selection of therapeutic modalities, for evaluation of medical, interventional and surgical therapies and for prediction of acute coronary syndromes.

In 1999, dye-image coronary angioscopy was established by Y. Fujimori, my colleague, for identification of endothelial damage. Fluorescence-image coronary angioscopy was also performed clinically by the author (1999).

These new techniques may yield more detailed information on composition and metabolism of coronary plaques. Angioscope-guided transcatheter interventions which were applied to peripheral vessel diseases (1992) may also be applied to the coronary artery in the near future. Now, angioscopes for coronary use contain more than 8000 glass or silica fibers which may give us much more improved pictures of coronary liminal changes. Angiomicroscopy, which enables evaluation at the cellular level applied to peripheral vessels, (1995) may also be applied to coronary arteries if it becomes flexible.

Observation of coronary microvessels is beyond conventional angioscopy. One possible approach for observation of coronary microvessels is their observation from the inside of the cardiac chambers.

In 1912, von Rhea and Walker observed the interior of the heart during thoracotomy. In 1922, D.S. Allen, and in 1944, D.E. Harken also observed the interior of the heart during thoracotomy in animals. In 1948, by using a rigid endoscope, S. Sakakibara and his colleagues observed the interior of the right heart during open heart surgery. This may be the first observation of the interior of the heart in humans. However, many years passed before percutaneous cardioscopy was performed for observation of the interior of the beating heart in patients, due to a lack of thin fiberscopes and effective equipment for displacement of blood.

In 1988, the authors successfully observed percutaneously the right and left ventricles from the inside using a thin fiberscope incorporated in a balloon-guided catheter in anesthetized dogs, and it was applied in patients with heart disease in 1988. Application of this "Cardioscopy" was extended to dilated cardiomyopathy, myocarditis, hepertrophic cardiomyopathy and ischemic heart disease, and for evaluation of drugs on subendocardial microcirculation (1995). In addition, cardioscope-guided endomyocardial biopsy was established (1990). At present, dye-image and fluorescence-image cardioscopy is clinically attempted. Cardioscope-guided intramyocardial and intrapericardial drug administration was started recently.

In 1996, intracardiac ultrasonography (ICUS) was also established and it is routinely used clinically for evaluation of cardiac chambers and valves. Combined use of cardioscopy and ICUS gives us much more information on cardiac wall architecture.

On April 1, 2000, The Japanese Association for Cardioangioscopy was established based on 13 years' preceding activities on cardioangioscopy. Cardioangioscopic examination is now supported by the national health insurance in Japan. This modality of examination will be used for diagnosis as well as for guidance of intracardiac and intravascular therapies in the near future.

Yasumi Uchida
Clinical Physiology and Cardiovascular Center
Toho University Hospital at Sakura
Guest Editor

